# Some Observations on Blood Diseases

**Published:** 1866-01

**Authors:** Edward B. Stevens

**Affiliations:** Cincinnati


### SOME OBSERVATIONS ON BLOOD DISEASES.

By EDWARD B. STEVENS, M.D., of Cincinnati.

[From the Transactions of the Ohio State Medical Society.]

It is not the purpose of the present paper to present a systematic essay on those diseases which are supposed to be associated with a poisoned condition of the blood; but rather such reflections on the whole subject as may serve to present with fairness some of the most prominent points of doctrine now entertained. To go beyond this would require a maturity of investigation that few of us dare aspire to. Indeed, the more carefully we pursue our researches upon these points, the less inclined we are to express our opinions dogmatically.

Two extreme schools in medical opinion have always prevailed as to the general pathology of morbid impressions; and two extremes of therapeutics, to a certain extent, correspond with our pathology; and thus, as we lean in our educational prejudices, to *humoralism* or *solidism*, we are very apt to become wedded to ideas of one or the other direction of thought, provoking a disposition to make light of all views not set to our peculiar theory, and rendering us forgetful of that which now seems the most satisfactory view, that there are varied means of receiving morbid impressions, and that there are varied directions toward which we should apply our therapeutical agencies.

In the present state of our physiology, we suppose the constitution of the blood, its office, and the supply of its elements are pretty generally agreed upon. Through the medium of that fluid, nutriment for the general supply of the system and for the constant repair of each individual tissue is steadily afforded. The blood not only is the machine which matures and prepares these elementary materials, it also is its own disbursing agent, whose unerring instinct bestows upon each needy individual exactly its requisite material.

Now, in health there is a tendency to a certain consent standard, of uniform constituency in the composition of the blood, and yet various circumstances may modify the relative proportion of its elements to a certain extent, without producing any manifest effect on the state of the organism which can really be regarded as pathological. Diet, exercise, hunger, exposure, and a variety of constantly changing circumstances, may give a large amount of red corpuscles, or an increase of fibrin, or an excess of water, etc., etc., and yet the health of the individual not undergo any speedy marked detriment. There would seem to be certain, not very clearly understood, compensating elements of control in the system, which for a time enables it to resist these constantly occurring vibrations from what might be supposed to be a healthy standard.

On the other hand, we are remarkably impressed with the fact of how slight a change in the normal arrangement and proportion of the elements of the blood may initiate the most serious morbid processes, and secure the most fatal results.

Furthermore, it is to be borne in mind that not all deviations from a normal standard of the blood, associated with morbid conditions of the system in any of its organism, are by any means to be regarded as *true blood diseases.* That the blood is lacking in its proper elements, or there is a disproportion in their arrangement, or there is a foreign element present, does not necessarily imply that the deceased condition of the system is the result. The state of the blood may happen as a regular link in the chain of morbid processes otherwise progressing; and here is apt to be one fault that from one instance or group of facts, we are so prone to generalize. The remark, however, suggests to us to look at the blood as a diseased structure from several aspects; and we shall do this all the more satisfactorily to ourselves if we fortunately happen to have no established theories to control *us*, or be themselves unsettled.

(*Food.*) In our reflections upon this subject, we are by no means to forget the relation of food to the blood condition; for while it is true that very many apparently and actually diverse elements of diet seem to afford a sufficiently uniform state of the blood, yet the instinctive capacities of appropriation and assimilation will not meet every embarrassment. The bee hovers over the sweetest clover fields and the most filthy cesspool, and her honey sac is yet abundant in its pure secretion. So, too, the digestive apparatus of man elects from a wonderful variety of substances the elements which finally become converted into the uniform blood structure. Nevertheless, we observe after all that certain laws do hold, and will not suffer to be lightly regarded or trifled with.

Thus, for example we find that there is a necessity for regular supplies of fluid. If a man be entirely deprived of water for eight or ten hours, there follows exhaustion in a marked degree, while he may pass the same length of time without solid food without any serious or special inconvenience. So, too, the experiments of physiologists have shown that animals supplied alone with water live several days longer than those supplied alone with solid food. We do not propose in the present paper to discuss the philosophy of these facts, but simply allude to them as links in the chain of facts, illustrative of the vitality afforded through the blood-status; but this being the recognized vehicle whereby nutriment finds its ways to the tissues no circumstances must be permitted to vitiate its elements. *Chloride of sodium* is one of the inorganic materials which require to be afforded with very considerable regularity; and certain well known pathological conditions are manifested with the deprivation. In the natural history of this food question, the natural craving of the human species for vegetable food, whereby the starchy or saccharine elements are afforded, as also the desire for oleagenous food, becomes highly suggestive, taken in connection with the reflections we are engaged in considering. So far as the craving for vegetables is concerned, it is found that even in certain pathological states, as for example in diabetes where a strictly animal diet has been persisted in as a means of therapeutics, yet very soon the instinctive craving becomes too imperative to be overlooked, and the treatment is abandoned.

Certain products of the blood, which we must confess, however, are not very well understood, are materially influenced by the character of the food. Of these are urea, the urates, carbonic acid, and the excretions generally. And indeed this is true whether we regard these as products of the blood direct, or results of disintegration, or whatever theory we may adopt.

Now, bearing in mind these well known elements of a healthy blood constitution, crudely expressed as they are, it requires no elaboration of facts or illustration to suggest the subtle influence of improper diet, or want of diet, or irregular diet, of bad air, of deranged or arrested eliminating function, together with an endless list of occurrences or circumstances which will by some of its processes materially change the blood in one of at least three ways: viz., an excess of natural elements, a loss of natural elements, the introduction of various unusual or foreign elements.

Either of these conditions of abnormal constitution are manifestations of blood disease, or speedily provocative of blood disease.

In the whole range of therapeutical agents, we have no more interesting group of study than those remedies which from their supposed peculiar action are styled *Restoratives* and *Catalytics.*

That a great variety of medicines, when administered, produce more or less important modifications in the blood, seems now too well established to require any elaboration in such a paper as this. Whether they act through the blood directly or by virtue of some indirect sympathetic influence is also immaterial to our present purpose. What we most wish to know is that medicines do change the character of the blood and through this structure affect all the other structures of the system. Mr. Headland, in his excellent book, regards all these remedies as acting in the blood, that they pass into the blood, and produce their influence there. He styles them *Hœmatics.*

It is certainly not very difficult to understand that a great many diseases arise simply from a want of some one or more of the normal constituents of the blood. That this is the case in simple debility, would appear plain enough. We are quite agreed that anæmia occurs from a deficiency of the hæmatosin of the blood corpuscles. Certain diseases are associated with a lack of the salts of potash. In malignant cholera there is an absence of the watery particles of the blood. Some forms of urinary disease are manifested by their peculiar urinary deposits—here there is probably a deficiency in the elements of the blood whose office is to hold these elements in solution. We have thus suggested a wide range of pathological condition, in which there is some defect in the blood elements. Medicines which supply this defect serve to restore a correct condition of the faulty functions, hence we style them Restoratives. We must not in this connection lose sight of the distinction—all this class of agencies are not in any sense eliminators of any vice—they do not necessarily pass out of the blood, they afford to it something wanting from its healthy condition. And in this sense, *articles of food* become in the highest sense restoratives. They stand at the head of the class, and with the whole train of hygienic influences, are our first resources.

Some years ago, a distinguished fellow member of this Society read a Prize Essay to this body on *Essential Fevers*, in which he elaborated very fully and clearly the doctrine of foreign elements as the origin of certain putrid fevers. It is known that the normal condition of the blood is alkaline. Carbonate of soda is one of the healthy constituents, and recently Dr. B. W. Richardson has pretty well established the coagulability of the blood as dependent on the escape of ammonia (or its carbonate.) Now there is very fair reason to believe that typhoid fever, and other low forms of fever, especially all that class of fevers known as "putrid fevers" are dependant on an excess of some of these alkaline elements. Thus, for example, Dr. Blair has shown that in the yellow fever there is an excess of the alkali of the blood, and that this alkali is the ammonia. At any rate, therapeutically there it no doubt but acids are of marked service as remedies in the treatment of these fevers, as the empirical experience of the profession ascertained long ago. More than a hundred years ago (in 1750,) Huxham recommended the mineral acids in the treatment of "putrid crasis" in fevers. And in our time a favorite treatment of scarlatina is the administration of nitric acid.

There is some dispute whether the acid of the gastric juice be the hydrochloric or lactic, the former is generally supposed. Now in many cases of weak digestion the administration of an acid has acted as a stimulous or promoter of the function. The explanation seems to be that a larger amount of acid is set free in the blood, thereby counteracting the failure of gastric secretion upon which these dyspeptic conditions depend. For similar reasons some forms of diarrhœa are relieved by acids.

Similar processes of reasoning are applicable to many other natural elements of the blood, and their naturally suggested restoratives, as alkalies, tonics, preparations of iron, and perhaps some other classes of agents.

"When a disease depends on the want of some material in the blood system, then it admits of being cured by a restorative. When a morbid process results in a diminution of the amount in the blood of some necessary constituent, then also may a restoration be of use in alleviating the consequences of such a disorder."—*Headland, Hœmatics.*

There is another branch of this subject, containing a proposition, to which the profession has given its assent, after some mode, through all ages; and yet for which there is even to this day no very definite demonstration, and to which there is, with many eminent authorities of medicine, the most virulent unrelenting skepticism and warfare.

It is common to ascribe to certain peculiar remedies certain important specific offices in the human economy, for example, *mercury* is, after all that has been said, still esteemed as the antagonist of syphilis; arsenic of lepra; and iodine of scrofula. We have never been able yet to say how these medicines produce their specific action, we simply know that they have the power to antagonize particular pathological conditions.

In the obscurity which has ever surrounded the *modus oper-*

*andi* of these and other remedies, it has been the theory to regard them as acting through the blood. That certain *materies morbi* are working in the blood, of which the vital tendency is to pass out by the gradual processes of functional elimination, and that for particular forms of foreign matters thus existing in the blood, there are respectively certain appropriate antagonizing agents. Now, we say that the demonstrations of these subtle processes are by no means clear, yet it is difficult otherwise to satisfactorily account for an extended list of phenomena in the natural history of disease and therapeutics.

In the valuable prize essay of Prof. Armor, already referred to in this paper, the class of diseases resulting from these peculiar toxic conditions of the blood are styled zymotic, and writers generally have used this term, and remedies which antagonize these poisons are spoken of as *catalytics.*

Now I say there has been some very similar way of expressing this idea from the earliest times to now. What a wonderful similarity in the old doctrines of Hippocrates, his coction or fermentation, and critical days, with the fancy of Liebig, who compares the history of these blood disorders to the process which takes place when a small quantity of yeast is introduced into a mass of malt (or sweet wort.)

Yeast, as every scientific person knows, is a vegetable fungus; placed in a solution of sugar, it undergoes rapid development, and as a consequence, alcoholic fermentation takes place, the elements of sugar arrange themselves in the new and simple forms of alcohol and carbonic acid; but in the malt there is another element, the gluten; and this gives rise to another peculiar manifestation, the yeast multiplies itself enormously at the expense of the gluten, and when the process is completed there is twenty or thirty times as much yeast as there was placed in the malt at the beginning. Now, Liebig uses this process to illustrate the events which take place when a toxic element finds its way into the blood. He believes that by some mysterious operation resembling (*not the same,*) but resembling the yeast fermentation and growth, when a portion of the poison of small-pox, or scarlatina, or diphtheria, or syphilis finds a lodgment in the blood it may so act upon the usual elements, or upon some peculiar elements of that fluid, as to reproduce itself indefinitely. To effect this reproduction, just as the yeast requires the gluten, the toxic element must have its elementary affinity present upon which to react, otherwise there is no increase, there may or may not be some disturbance of the economy, and there is by and by elimination. If the required element be present, the process goes on, and new relations are established kindred to fermentation; in proportion to the importance of this element, as a material of tissue life and growth, will be the degree of disturbance produced; if the element is concerned in vital operations, then the toxic condition overwhelms the system and proves more or less rapidly fatal, as we are to suppose, according to the concentration of the poison.

The doctrine of typical changes so beautifully elaborated by Mr. Paget, is really only another way of expressing a similar theory. He likens the process of change which occurs in the pathological history of a typhus fever, or a small-pox, or any of those, what are styled zymotic diseases, to the changed formative process seen in a scar, or other changed tissue. A new elementary disposition has occurred, a new type, and thereafter the formative process proceeds after the new order, and hence as he supposes, the immunity thenceforth, to the influence of this toxic element if reintroduced into the blood; that is to say, the gluten type of Mr. Liebig has been destroyed and the new formative process ceases to produce it. So, too, Simon and other eminent pathologists present their special explanations of the events which we know to occur, and when analyzed, there is still the old "crisis," and "coction," and "humoral poison" of that great philosophor and physician, Hippocrates.

The pertinacity with which these ideas have retained their hold on the philosophy of medicine suggests a large degree of respectful attention, as if not absolutely true or demonstrated to be true, yet as probably on the right track.

These ideas have naturally suggested many important therapeutical views and experiments, of great value practically. The idea of a toxic condition at once suggests its antagonist; and when, on the one hand, we observe the natural history of acute rheumatism to require a definite period, expressed by Abernethy as "six weeks," to pass through its process of coction, its crisis, and its decadence, oftentimes this natural history indefinitely extended, with its incidents of chalky deposits and the like, *when undisturbed.* And on the other, when a course of alkaline medication on the plan of Mr. Fuller has arrested the process of disorder in a few days, we find it very difficult to divest ourselves of a corresponding theory.

In like manner we find very strong circumstantial evidences of toxic processes in such diseases as erysipelas, uræmic intoxication, septicemia, and the like. Exactly what blood changes have taken place in tubercle and scrofula; in putrid fevers; in the lengthy catalogue of cutaneous diseases, perhaps we shall not be permitted to know. But the therapeutical suggestions and experiences which are accumulating in this field of inquiry are most fascinating in interest, and of the highest practical importance. It had been our purpose to embrace the consideration of some of these topics in the present paper, but the *resume* of the points already submitted have drawn more on the patience of the Society than was expected, and if agreeable, it will be my pleasure on a future occasion to prepare a supplementary paper, embracing some review of the more important topics to be considered under the general idea of *The Recent Therapeutics of Zymosis* or *Catalytic Diseases.*

